# Editorial: From sea to fork: novel seafood and effects on human health

**DOI:** 10.3389/fnut.2023.1339216

**Published:** 2023-12-01

**Authors:** Alicia del Carmen Mondragon Portocarrero, Carmen Adriana Campos, Jose Manuel Miranda Lopez

**Affiliations:** ^1^Laboratorio de Higiene, Inspección y Control de Alimentos (LHICA), Departamento de Química Analítica, Nutrición y Bromatología, Facultad de Veterinaria, Universidade de Santiago de Compostela, Lugo, Spain; ^2^Departamento de Industrias, Instituto de Tecnología de Alimentos y Procesos Químicos (ITAPROQ), Consejo Nacional de Investigaciones Científicas y Técnicas (CONICET), Buenos Aires, Argentina

**Keywords:** seafood, seaweed, algae, marine lipids, frailty, prebiotic, food allergies

Since ancient times, the sea has been an essential source of food for humans. In the sea, a large plethora of foods provide humans with benefits for health, such as *n-*3 fatty acids, proteins of high biological value, or essential minerals such as zinc (1–3). High seafood consumption has been associated with improved physiological functions and the prevention of several diseases (1, 3).

Although seafood is usually consumed worldwide, it is more commonly consumed within dietary patterns that are traditionally positively associated with health and longevity, such as the Mediterranean diet (4), the Atlantic diet (5) or the traditional Japanese diet (6).

Currently, the production of terrestrial foods has numerous problems that hinder its production. In fact, under the pretext that CO_2_ is responsible for global warming, highly questionable affirmation from a scientific point of view, some political authorities, such as the European Union, have launched a crusade against agriculture and livestock farming that severely limits their production capacity. However, in the context of a continuously expanding world population, it is expected that in the next decades, there will be a need to increase food production to ensure the food supply of the entire population (7). In this sense, it is projected that in 2050, it will be necessary to increase world food production by 35%−56% (7). Considering these factors, the exploration of alternative and complementary foods that are sustainable, nutritious, and affordable becomes crucial (Wu et al.).

In this sense, the capacity of the sea to provide us with beneficial foods and nutrients has not been fully explored (3). Thus, the aim of this Research Topic is to update the knowledge about novel uses or effects on the human health of seafood. Included in this Research Topic, three original articles and three reviews were published.

Ahn et al. demonstrated that the consumption of seafood, particularly fish, could be beneficial for preventing frailty in Korean community-dwelling older adults. These effects seem to be exerted through the modulation of the *n-*3 PUFA content in the membranes of erythrocytes, which was inversely associated with the prevalence of frailty in older people. Another research article conducted by Lee et al. found that fucoidans extracted from brown seaweeds by hydrothermal extraction can be a good alternative to reduce human retinal pigment epithelium cells and thus contribute to the management of ocular tissue. In this sense, seaweeds contribute to improving health through the use of their bioactive compounds as nutraceuticals.

The consumption of seaweeds as food products or functional ingredients in the diet of Western countries has been progressively increasing (2). Lopez-Santamaria et al. demonstrated that brown seaweeds, such as *Saccharina japonica* and *Undaria pinnatifida*, had prebiotic effects when consumed as whole seaweeds.

Two reviews have focused on the future role of algae (including macro- and microalgae) as future sources of protein for human food. Algae are an interesting source of protein due to their high growth rate, high photosynthetic efficiency, and low water consumption, and they do not require land for growth (Espinosa-Ramírez et al.). Although research on the use of algae in the development of meat alternatives is at an initial stage, promising results could be expected soon due to their diverse composition of bioactive and technological molecules (Espinosa-Ramírez et al.). Additionally, the biological effect of algae impacts metabolic health due to glucose and lipid homeostasis as well as anti-inflammatory properties (Wu et al.).

Finally, another review addressed the subject that dietary marine lipids can also modulate the gut microbiome (Abril et al.). An important number of the physiological effects of marine lipids are induced by the incorporation of eicosapentaenoic acid and docosahexaenoic acid into the phospholipid membranes of cells. In this way, the structure and composition of the gut microbiome would be modulated, promoting human health benefits, such as in the case of food allergies (Abril et al.).

In summary, by examining the articles included in this Research Topic, readers can gain a better understanding of the relevance for human health and future potential of seafood.

## Author contributions

AM: Writing—original draft. CC: Writing—original draft. JM: Conceptualization, Writing—review & editing.
